# From evolutionary individuality to cognitive integration: a cross-modular perspective on human–AI coevolution

**DOI:** 10.3389/fpsyg.2026.1768785

**Published:** 2026-09-18

**Authors:** Edward Ruoyang Shi

**Affiliations:** Tianjin University of Finance and Economics Pearl River College, Tianjin, China

**Keywords:** cognitive individuality, cross-modular integration, episodic memory, human–AI coevolution, Neo-UnCartesian linguistic program, representational architecture

“*Complex minds can't develop on their own. [...] For a mind to even approach its full potential, it needs cultivation by other minds.”*

—Ted Chiang, The Lifecycle of Software Objects (2010)

## 1 Introduction

Rainey and Hochberg (2025) extend the framework of Major Evolutionary Transitions (METs) to human–AI relations. B0y comparing increasingly tight human–AI coupling with canonical transitions such as chromosomes, eukaryotic cells, and multicellularity, they ask whether cooperation, division of labor, and aligned interests could eventually produce a new evolutionary individual. This is a productive question because it relocates discussion from isolated machine capability to organization across scales (Smith and Szathmary, 1997). The present article does not replace that framework or criticize it for failing to answer a different question. It offers a supplement: even if a human–AI composite acquired some properties of an evolutionary individual, what additional organization would make it a cognitive individual?

Individuality has no single accepted currency. Biological and philosophical accounts emphasize, among other things, reproduction and selection, operational closure, autonomy, informational integration, temporal persistence, and organism–environment partitioning (Maturana and Varela, 2012; Godfrey-Smith, 2009; Friston, 2013; Krakauer et al., 2020). These criteria are relevant to human–AI composites. Yet none by itself determines whether heterogeneous information is organized into contents that can guide perception, action, inference, and evaluation across time. I therefore treat representational architecture as a domain-specific criterion of cognitive—not biological—individuality. It is privileged here because the target phenomenon is meaningful, flexible cognition: the capacity to preserve referential relations, recombine event content, compare alternatives, and revise commitments. This is a theoretical choice rather than a claim that all cognition must be explained representationally.

The qualification matters. Enactive, ecological, 4E, and distributed approaches correctly emphasize that cognition depends on bodily action, environmental structure, and dynamic coupling rather than on a self-sufficient inner model (Varela et al., 1991; Hutchins, 1995; Chemero, 2013). The present proposal is compatible with their anti-isolationist lesson. Its narrower claim is that flexible human cognition also depends on structured internal states whose content and accessibility are transformed through interactions among partially specialized systems. Accordingly, “unified internal model” should not mean a complete, centralized picture of the world. It means a sufficiently coherent and revisable organization of representations that preserves relations across domains and temporal intervals while remaining embodied and environmentally scaffolded.

This yields the article's central distinction. Functional integration occurs when components reliably contribute to joint outcomes. Cognitive integration, in the stronger sense proposed here, occurs when their recurrent interaction changes what the composite system can maintain, access, and revise as a representation. Functional coupling can therefore be deep without being constitutive; conversely, constitutive integration is gradual rather than all-or-none. The relevant question is not simply whether an AI affects a human decision, but whether information crosses the interface bidirectionally, is reformatted for multiple cognitive systems, persists as part of a common temporal history, and is answerable to the human agent's goals and error-correction practices.

Human language illustrates this architecture. Syntax provides a generative and hierarchical format in which elements can enter nonlocal dependencies and be recombined beyond the immediate situation. Event cognition and episodic memory bind agents, actions, objects, locations, goals, and temporal relations into structured situations (Tulving, 2002; Zacks et al., 2007; Radvansky and Zacks, 2017). Semantic cognition recruits widely distributed modality-specific and heteromodal systems rather than a single store (Binder and Desai, 2011). Linguistic meaning is thus supported by interactions among grammatical structure, conceptual knowledge, event models, perception, and memory (Zwaan and Radvansky, 1998; Ferstl et al., 2008). The syntax–episodic memory interface proposed in Shi (2024, 2025a,b, 2026a) represents one specific hypothesis within this broader independent literature, rather than the sole basis of the present argument (see also Shi, 2026b; Shi et al., 2026; for related discussions from the perspective of the Neo-UnCartesian Linguistic Program [NULP]).

## 2 Cross-modular architecture and cognitive individuality

### 2.1 What “cross-modular” means

“Cross-modular” is used here in a deliberately restricted sense. It does not assume classical Fodorian modules that are wholly encapsulated, nor massive modularity, nor a globally undifferentiated network. It begins from partial specialization: linguistic structure, episodic-relational memory, perception, affect, and social cognition operate with different formats, timescales, and computational constraints. Integration is cross-modular when recurrent processes (i) translate or index content across at least two such formats, (ii) allow the translated content to constrain subsequent processing in both systems, and (iii) preserve enough identity across transformations for the content to be retrieved and revised. Mere co-activation or serial transfer of an output does not satisfy this definition.

A cross-modular representational loop can therefore be identified through four diagnostic properties. First, bidirectional constraint: event knowledge affects linguistic construction, and linguistic structure reorganizes event retrieval or prediction. Second, persistence: the linked representation remains available beyond the immediate exchange. Third, cross-domain accessibility: the same represented event can guide memory, inference, action, affective appraisal, or communication. Fourth, recursive revision: conflict or prediction error can update the representation and alter later processing. These are graded empirical criteria. They can be investigated through behavioral transfer, representational-similarity analyses, effective connectivity, lesion dissociations, and longitudinal changes in coupled systems.

### 2.2 Syntax, events, and referential continuity

The proposed loop assigns complementary—not exhaustive—roles to syntax and episodic memory. Syntax provides combinatorial form but is referentially underspecified on its own. Episodic memory provides relational and temporal organization but does not by itself supply the open-ended grammatical formats required for linguistic displacement and recombination. Event models bridge perception and memory by segmenting continuous experience into hierarchically organized units that support prediction and later recall (Zacks et al., 2007; Baldassano et al., 2017). Discourse comprehension similarly requires readers to update situation models along dimensions such as time, space, causation, protagonists, and goals (Zwaan and Radvansky, 1998). Hippocampal–cortical interactions contribute relational binding and constructive simulation, while semantic and language networks provide conceptual and combinatorial resources (Ranganath and Ritchey, 2012; Schacter et al., 2012; Duff and Brown-Schmidt, 2012).

Meaning therefore does not arise from episodic memory alone, and reference is not reducible to first-person experience. Semantic memory, social conventions, perceptual grounding, and inferential practices all contribute. The narrower claim is mechanistic: event indices help linguistic structures maintain who did what to whom, where, and when across clauses and episodes. Recurrent coupling among these systems supports counterfactual recombination and narrative continuity. Autobiographical memory, for example, is organized through interactions among episodic specificity, conceptual self-knowledge, and life narratives rather than through a simple archive of experiences (Conway, 2005). This broader architecture makes it possible for represented events to be evaluated from affective and social perspectives.

### 2.3 Why this supplements MET theory

MET theory and the present account address different explanatory levels. Selection-based or informational criteria ask when lower-level entities form a higher-level evolutionary unit. Cross-modular criteria ask when components jointly realize an architecture capable of maintaining and revising cognitive content. Evolutionary individuality can therefore occur without cognitive individuality—as in many biological collectives—and cognitive integration can increase without producing a new unit of selection. In the human–AI case, cooperation and informational dependence are necessary evidence of a changing system, but they do not settle whether a new center of representation and control has emerged.

The emergence of language illustrates the possible bridge between the levels without reducing one to the other. Language altered the ecological and social conditions of cooperation while also reorganizing interfaces among memory, perception, action, and social inference. The cognitive transition is not defined primarily by heredity or system–environment partitioning, but by new representational operations made available to the organism and its group. Thus, the cross-modular proposal does not redescribe every MET as a representational transition. It specifies an additional criterion for transitions whose explanandum is cognitive individuality.

## 3 Levels of human–AI integration

The contrast between a tool and a new cognitive individual should not be treated as exhaustive. Human–AI relations can deepen along several partially independent dimensions: reciprocity, persistence, personalization, informational dependence, cross-domain accessibility, and control. The following taxonomy integrates these dimensions while reserving constitutive cognitive integration for the strongest case (Table 1).

**Table 1:** A graded taxonomy of human-AI integration.

Level	Defining relation	Diagnostic criterion	Typical status
1. Instrumental use	External, task-specific use	AI output can be removed without reorganizing the user's standing cognitive routines.	Current tools
2. Cognitive scaffolding	AI organizes, retrieves, or reformats information	Reliable support improves a human process, but goals, evaluation, and continuity remain human-maintained.	Common today
3. Recurrent functional coupling	Stable, reciprocal routines develop across repeated interaction	Each partner's next operation is regularly conditioned by the other; the routine persists over time.	Emerging
4. Partial informational integration	System-level state becomes historically interdependent	Shared records and feedback make future human–AI states depend on their interaction history, with some mutual error correction.	Possible in agents/workflows
5. Constitutive cognitive integration	A shared representational architecture participates in cognition	Event representations are jointly maintained across time, accessible across domains, recursively revisable, and integrated with human goals and normative evaluation.	Not established

The table distinguishes five analytically separable levels of human-AI relations-from instrumental use and cognitive scaffolding to recurrent functional coupling, partial informational integration, and constitutive cognitive integration-according to their defining relations, diagnostic criteria, and current technological status. These levels do not constitute an inevitable developmental sequence, and a given system may exhibit characteristics associated with more than one level.

The levels are analytic rather than an inevitable developmental sequence. A system may show strong informational dependence yet weak normative control, or persistent personalization without cross-domain accessibility. The taxonomy also clarifies why frequent use alone is insufficient. A navigation system may become indispensable to performance while remaining an external scaffold; a long-running agent may preserve interaction history without integrating it into a human user's autobiographical organization. Movement toward constitution requires not only tighter coupling but a change in the representational economy of the combined system.

## 4 Human–AI coevolution through a cross-modular lens

### 4.1 Existing achievements and their limits

Large language models are not confined to simple surface matching. They learn rich distributional regularities and can support planning, retrieval, and structured generation. Moreover, current research explicitly adds memory stores, multimodal perception, embodied action, world models, and continual-learning mechanisms. Generative agents, for example, store records of experience and retrieve and synthesize them to guide later behavior (Park et al., 2023); world-model research learns temporally structured latent dynamics for prediction and control (Ha and Schmidhuber, 2018; Hafner et al., 2023). These developments justify rejecting the categorical claim that AI has no event-like or temporally organized representations.

What remains unestablished is equivalence to human episodic memory or participation in a shared human–AI perspective. Artificial memory is often externally engineered, task-bounded, and optimized for retrieval or control. Its records need not be organized by autonoetic awareness, affective salience, embodied vulnerability, or autobiographical continuity. Likewise, multimodal grounding can connect symbols with sensory arrays without creating a single, enduring representational history across the human and artificial components. The limitation is therefore comparative and architectural, not an assertion that artificial systems are stateless or incapable of temporal modeling.

### 4.2 From scaffolding to constitution

The extended-mind literature offers criteria for evaluating intermediate cases. Clark and Chalmers (1998) emphasize reliable availability and automatic endorsement; cognitive-integration approaches further stress complementarity, transformation, and the emergence of a coupled process rather than simple substitution (Menary, 2007; Heersmink, 2015). Applied to AI, these criteria suggest that an external component becomes more constitutive as interaction becomes reciprocal, reliable, personalized, transparent enough for correction, and integrated into ongoing tasks and identity-relevant practices. Yet reliability and dependence are not sufficient for the strongest claim made here. A constitutive cross-modular component must also participate in maintaining event relations across time and permit those relations to constrain multiple domains of cognition.

This formulation makes the proposal falsifiable. Evidence for stronger integration would include stable cross-session event representations jointly accessible to human and artificial processes; bidirectional updating in which human recollection changes the artificial record and artificial retrieval measurably reorganizes later human recall or inference; transfer of jointly maintained content across linguistic, perceptual, planning, and affective tasks; and error-correction procedures that preserve human authorship and control. Evidence of dependence without these properties would support recurrent coupling or partial informational integration rather than a new cognitive individual.

### 4.3 Meaning, agency, and normativity

Episodic grounding should not be treated as sufficient for meaning or moral responsibility. Normative agency also requires capacities for control, intention, responsiveness to reasons, and participation in social practices of attribution. Event-grounded continuity matters because responsibility concerns actions by agents situated in histories, but it is one condition among several. Current AI systems may influence morally significant decisions while remaining objects of governance rather than bearers of responsibility. Accountability should therefore remain with human and institutional agents unless there is independent evidence for the relevant forms of control and normative competence.

The constructive goal is consequently not to simulate a machine autobiography or prematurely attribute consciousness. It is to design interfaces that improve human integrative capacity while keeping provenance, revision, and authority visible. Artificial systems could help users organize episodic material, explore counterfactuals, and detect inconsistencies, but the architecture should preserve the user's ability to accept, reject, reinterpret, or delete machine-supported representations. Such designs deepen scaffolding without obscuring where normative responsibility lies.

## 5 Normative implications and research agenda

A graded account changes the normative question. The risk is not only that AI will remain external, nor only that it will become autonomous, but that systems will occupy intermediate levels while being represented as either mere tools or genuine partners. Recurrent coupling can reshape attention, memory, and judgment even without constitutive integration. Partial informational integration can also create path dependence: errors in a persistent record may influence future retrieval, decisions, and self-description. Evaluation must therefore track both benefits and vulnerabilities at each level.

Three research priorities follow. First, human–AI studies should measure representational change, not only task performance: does repeated interaction reorganize event segmentation, recall, confidence, or narrative interpretation? Second, system design should separate storage, inference, and normative authorization so that jointly produced representations remain inspectable and corrigible. Third, longitudinal research should examine whether stable coupling produces cross-domain transfer and temporal continuity, while distinguishing genuine integration from automation bias, dependency, or simple offloading. These questions connect evolutionary scale, neural and cognitive mechanism, and linguistic representation without collapsing them into one vocabulary.

## 6 Conclusion

The MET framework identifies an important possibility: sustained human–AI cooperation may reorganize individuality at evolutionary and informational levels. A theory of cognitive individuality adds a distinct question—whether the composite maintains and revises representations as an integrated system. The proposed cross-modular criterion locates strong cognitive integration in recurrent, bidirectional, persistent, cross-domain, and corrigible interactions among generative structure, event memory, perception, affect, and social evaluation. This criterion is representational but not isolationist: representations remain embodied, environmentally scaffolded, and socially governed.

Current AI systems already exceed mere instrumentality in some settings. Memory-augmented agents, multimodal models, and stable workflows can support cognitive scaffolding, recurrent coupling, and partial informational integration. What has not been demonstrated is constitutive participation in a shared, temporally continuous, event-grounded, and normatively revisable architecture. Human–AI coevolution should therefore be understood as a graded transformation whose decisive issue is not scale alone, but the organization, control, and revision of representations across the human–machine boundary.

## References

[B1] Baldassano C. Chen J. Zadbood A. Pillow J. W. Hasson U. Norman K. A. (2017). Discovering event structure in continuous narrative perception and memory. Neuron 95, 709–721. doi: 10.1016/j.neuron.2017.06.041 28772125 PMC5558154

[B2] Binder J. R. Desai R. H. (2011). The neurobiology of semantic memory. Trends Cogn. Sci. 15, 527–536. doi: 10.1016/j.tics.2011.10.001 22001867 PMC3350748

[B3] Chemero A. (2013). Radical embodied cognitive science. Rev. Gen. Psychol. 17, 145–150. doi: 10.1037/a0032923

[B4] Clark A. Chalmers D. (1998). The extended mind. Analysis 58, 7–19. doi: 10.1093/analys/58.1.7

[B5] Conway M. A. (2005). Memory and the self. J. Mem. Lang. 53, 594–628. doi: 10.1016/j.jml.2005.08.005

[B6] Duff M. C. Brown-Schmidt S. (2012). The hippocampus and the flexible use and processing of language. Front. Hum. Neurosci. 6:69. doi: 10.3389/fnhum.2012.00069 22493573 PMC3319917

[B7] Ferstl E. C. Neumann J. Bogler C. von Cramon D. Y. (2008). The extended language network: a meta-analysis of neuroimaging studies on text comprehension. Hum. Brain Mapp. 29, 581–593. doi: 10.1002/hbm.20422 17557297 PMC2878642

[B8] Friston K. (2013). Life as we know it. J. R. Soc. Interface 10:20130475. doi: 10.1098/rsif.2013.0475 23825119 PMC3730701

[B9] Godfrey-Smith P. (2009). Darwinian Populations and Natural Selection. Oxford: Oxford University Press. doi: 10.1093/acprof:osobl/9780199552047.001.0001

[B10] Ha D. Schmidhuber J. (2018). World models. arXiv [Pre-print]. arXiv:1803.10122. doi: 10.48550/arXiv.1803.10122

[B11] Hafner D. Pasukonis J. Ba J. Lillicrap T. (2023). Mastering diverse domains through world models. arXiv [Pre-print]. arXiv:2301.04104. doi: 10.48550/arXiv.2301.04104

[B12] Heersmink R. (2015). Dimensions of integration in embedded and extended cognitive systems. Phenomenol. Cogn. Sci. 14, 577–598. doi: 10.1007/s11097-014-9355-1

[B13] Hutchins E. (1995). Cognition in the Wild. Cambridge, MA: MIT Press. doi: 10.7551/mitpress/1881.001.0001

[B14] Krakauer D. C. Bertschinger N. Olbrich E. Ay N. Flack J. C. (2020). The information theory of individuality. Theory Biosci. 139, 209–223. doi: 10.1007/s12064-020-00313-7 32212028 PMC7244620

[B15] Maturana H. R. Varela F. J. (2012). Autopoiesis and Cognition: The Realization of the Living. Dordrecht: Springer Science and Business Media.

[B16] Menary R. (2007). Cognitive Integration: Mind and Cognition Unbounded. Basingstoke: Palgrave Macmillan. doi: 10.1057/9780230592889

[B17] Park J. S. O'Brien J. Cai C. J. Morris M. R. Liang P. Bernstein M. S. (2023). “Generative agents: interactive simulacra of human behavior,” in Proceedings of the 36th annual ACM symposium on user interface software and technology, Vol. 2, 1–22. doi: 10.1145/3586183.3606763

[B18] Radvansky G. A. Zacks J. M. (2017). Event boundaries in memory and cognition. Curr. Opin. Behav. Sci. 17, 133–140. doi: 10.1016/j.cobeha.2017.08.006 29270446 PMC5734104

[B19] Rainey P. B. Hochberg M. E. (2025). Could humans and AI become a new evolutionary individual? Proc. Natl. Acad. Sci. U. S. A. 122:e2509122122. doi: 10.1073/pnas.2509122122 40928882 PMC12452951

[B20] Ranganath C. Ritchey M. (2012). Two cortical systems for memory-guided behaviour. Nat. Rev. Neurosci. 13, 713–726. doi: 10.1038/nrn3338 22992647

[B21] Schacter D. L. Addis D. R. Hassabis D. Martin V. C. Spreng R. N. Szpunar K. K. (2012). The future of memory: remembering, imagining, and the brain. Neuron 76, 677–694. doi: 10.1016/j.neuron.2012.11.001 23177955 PMC3815616

[B22] Shi E. R. (2024). Across the boundary: the formalization of the interface between episodic memory and narrow syntax computation of human language. Biolinguistics 18, 1–24. doi: 10.5964/bioling.14649

[B23] Shi E. R. (2025a). How the brain recycled memory circuits for language: an evolutionary perspective on the ROSE model. Cogn. Neurosci. 16, 88–90. doi: 10.1080/17588928.2025.2561587 40964709

[B24] Shi E. R. (2025b). Beyond the cortex–integrating hippocampal function into the social brain hypothesis to explain advanced cognition. Behav.Brain Sci. 48:e182. doi: 10.1017/S0140525X25100472 41298092

[B25] Shi E. R. (2026a). Sequence as output, not limitation: comparative evidence for niche-driven language evolution. Evol. Linguist. Theory doi: 10.1075/elt.25002.shi

[B26] Shi E. R. (2026b). Binding events, building meaning: a new framework for pragmatic interpretation. J. Neurolinguistics 80:101359. doi: 10.1016/j.jneuroling.2026.101359

[B27] Shi E. R. Pei R. Barceló-Coblijn L. (2026). The digital therapist? LLMs and the future of clinical dialogue. Lang. Health. 4:100086. doi: 10.1016/j.laheal.2026.100086

[B28] Smith J. M. Szathmary E. (1997). The Major Transitions in Evolution. Oxford: Oxford University Press. doi: 10.1093/oso/9780198502944.001.0001

[B29] Tulving E. (2002). Episodic memory: from mind to brain. Annu. Rev. Psychol. 53, 1–25. doi: 10.1146/annurev.psych.53.100901.135114 11752477

[B30] Varela F. J. Thompson E. Rosch E. (1991). The Embodied Mind. Cambridge, MA: MIT Press. doi: 10.7551/mitpress/6730.001.0001

[B31] Zacks J. M. Speer N. K. Swallow K. M. Braver T. S. Reynolds J. R. (2007). Event perception: a mind–brain perspective. Psychol. Bullet. 133, 273–293. doi: 10.1037/0033-2909.133.2.273 17338600 PMC2852534

[B32] Zwaan R. A. Radvansky G. A. (1998). Situation models in language comprehension and memory. Psychol. Bullet. 123, 162–185. doi: 10.1037/0033-2909.123.2.162 9522683

